# Pharmacokinetics of CYP2C19- and CYP3A4-Metabolized Drugs in Cirrhosis Using a Whole-Body PBPK Approach

**DOI:** 10.3390/pharmaceutics17121582

**Published:** 2025-12-08

**Authors:** Ruijing Mu, Jingjing Gao, Xiaoli Wang, Jing Ling, Nan Hu, Hanyu Yang

**Affiliations:** 1 Center of Drug Metabolism and Pharmacokinetics, School of Pharmacy, China Pharmaceutical University, Nanjing 210009, China; juiching@stu.cpu.edu.cn (R.M.); jingjing199809@163.com (J.G.); 2 Mianzhu Municipal Health Bureau, Deyang 618200, China; wshelly001@163.com; 3 Department of Pharmacy, The Third Affiliated Hospital of Soochow University, Changzhou 213003, China; lingjing198888@126.com

**Keywords:** CYP2C19, CYP3A4, liver cirrhosis, physiologically based pharmacokinetic model, pharmacokinetics, dose optimization

## Abstract

**Background/Objectives**: Cirrhosis significantly alters physiological function and drug metabolism, particularly for medications primarily metabolized by CYP2C19 and CYP3A4. This study aims to establish a physiologically based pharmacokinetic (PBPK) modelling framework capable of predicting pharmacokinetic changes across different stages of cirrhosis, and to determine optimal dosing regimens that achieve drug exposure levels comparable to those in healthy individuals. **Methods**: We constructed a physiologically based pharmacokinetic (PBPK) model that incorporates six drugs, including omeprazole, lansoprazole, midazolam, ondansetron, verapamil, and alfentanil, which are metabolized primarily by CYP2C19 or CYP3A4. The pharmacokinetics of these drugs following oral or injectable administration were simulated in 1000 virtual healthy subjects, and the PBPK model was validated using clinical data. The model was further adapted to account for physiological changes in cirrhotic patients, extending its application to a population of 1000 virtual patients with liver cirrhosis. **Results**: Most observed data fell within the 5th and 95th percentiles of the virtual patient simulation results. Additionally, for most simulations, the area under the concentration-time curve (AUC) and peak concentration (C_max_) were within 0.5- to 2-fold of the observed values. Sensitivity analysis indicated that the reduced expression of metabolizing enzymes increased plasma concentrations of drugs, which was a major factor contributing to the elevated drug exposure in patients with cirrhosis. The clinical dosing regimens of the CYP2C19 substrate omeprazole and the CYP3A4 substrate ondansetron were optimized for use in cirrhotic patients. **Conclusions**: The developed PBPK model successfully predicted the pharmacokinetics of CYP2C19 and CYP3A4 substrates in both healthy individuals and cirrhotic patients and can be effectively used for dose optimization in cirrhotic populations.

## 1. Introduction

Globally, approximately 2 million people die each year from liver diseases, with half of these deaths attributed to cirrhosis and the remainder primarily resulting from viral hepatitis and liver cancer [1,2,3]. The World Health Organization (WHO) highlights cirrhosis as a major global health burden, responsible for over 1.4 million deaths annually, primarily driven by viral hepatitis, alcohol use, and nonalcoholic fatty liver disease [4]. As a progressive liver disease marked by architectural distortion and fibrosis, cirrhosis induces extensive physiological alterations that significantly influence the pharmacokinetics (PK) and pharmacodynamics (PD) of numerous drugs, thereby complicating clinical management [5,6].

The severity of cirrhosis is commonly assessed using the Child-Pugh (CP) classification system (grades A to C) [7,8], which provides insights into disease progression, evaluates surgical or medication risks, and informs treatment strategies. Cirrhosis induces a spectrum of physiological changes that collectively influence drug disposition. These changes include reduced hepatic blood flow, decreased hepatocellular enzyme activity, altered plasma protein levels, and impaired biliary excretion. Portal hypertension, a hallmark of cirrhosis, redirects blood flow away from the liver through portosystemic shunting, reducing the effective hepatic extraction of drugs. Additionally, the reduced functional hepatocyte mass diminishes the enzymatic capacity, particularly affecting phase I metabolism mediated by CYP enzymes [9]. Cirrhosis also leads to systemic physiological changes, including altered gastrointestinal absorption, renal function, and distribution volume of drugs [10]. Hypoalbuminemia, a common feature of cirrhosis, affects the protein binding of highly bound drugs, increasing their unbound concentrations.

Proton pump inhibitors (PPIs), such as omeprazole and lansoprazole, are extensively metabolized by CYP2C19, with a minor contribution from CYP3A4. Given their widespread use and narrow therapeutic window in certain populations, dose individualization is essential in patients with hepatic impairment. The 2020 guidelines issued by the National Health Commission of the People’s Republic of China recommend limiting omeprazole to ≤20 mg/day in patients with severe liver injury [11]. However, as this dose represents the standard therapeutic amount, such a generalized recommendation may be insufficient for managing interindividual differences among cirrhotic patients.

The cytochrome P450 enzyme system is highly sensitive to liver function impairment. CYP2C19, responsible for the metabolism of drugs such as PPIs and certain antiepileptics exhibits significant interindividual variability in cirrhotic patients [12,13] and plays a key role in converting clopidogrel into its active thiol metabolite; reduced CYP2C19 activity is strongly associated with clopidogrel resistance and an increased risk of stent thrombosis and other cardiovascular events [14]. CYP3A4, the most abundant hepatic CYP enzyme, metabolizes a wide range of drugs, including midazolam, cyclosporine, and statins, and its activity is regulated by both hepatic and extrahepatic factors such as intestinal metabolism. Consequently, CYP3A4 substrate drugs demonstrate significantly altered pharmacokinetics in cirrhotic patients, with midazolam showing markedly increased systemic exposure and prolonged sedation [15]. These pharmacokinetic alterations highlight the need for individualized dose adjustment and therapeutic drug monitoring for drugs metabolized by CYP2C19 and CYP3A4 [16,17]. Consistent with these observations, progressive liver injury has been shown to markedly downregulate hepatic metabolic enzyme expression [18]. In CP-C patients, hepatic CYP2C19 and CYP3A4 protein levels are reduced to 12% and 25%, respectively, of those in healthy individuals. Additionally, intestinal CYP3A4 expression is decreased approximately 3-fold. Such reductions in metabolic capacity substantially affect the in vivo disposition of affected drugs.

Physiologically based pharmacokinetic (PBPK) modeling has emerged as a powerful tool to predict the impact of cirrhosis on drug metabolism. By integrating physiological and biochemical data, PBPK models can simulate the altered pharmacokinetics of CYP2C19 and CYP3A4 substrates in cirrhotic patients. These models incorporate key parameters such as reduced hepatic enzyme activity, altered plasma protein binding, and changes in hepatic blood flow. Cirrhosis-induced physiological changes profoundly affect the pharmacokinetics of CYP2C19 and CYP3A4 substrate drugs, posing challenges to their clinical use. Understanding the pharmacokinetic alterations of these drugs is critical for optimizing therapeutic outcomes and minimizing adverse effects. PBPK modeling hold promise for improving the prediction of drug behavior in this population. Among published PBPK models [19,20] for liver injury, many include parameters that have been optimized based on clinical data, rather than being directly measured. Therefore, we attempted to adopt a bottom-up approach to develop the model using parameters from in vitro, and to incorporate as many relevant influencing parameters as possible in the modeling process to make it a highly predictive model.

In this study, we developed a PBPK model for PPIs, specifically omeprazole and lansoprazole, which are primarily metabolized by CYP2C19. The model was extended to simulate the pharmacokinetics of drugs metabolized by CYP3A4 in cirrhotic patients and to optimize drug dosing in this population.

## 2. Materials and Methods

### 2.1. General Workflow

**Figure 1:**
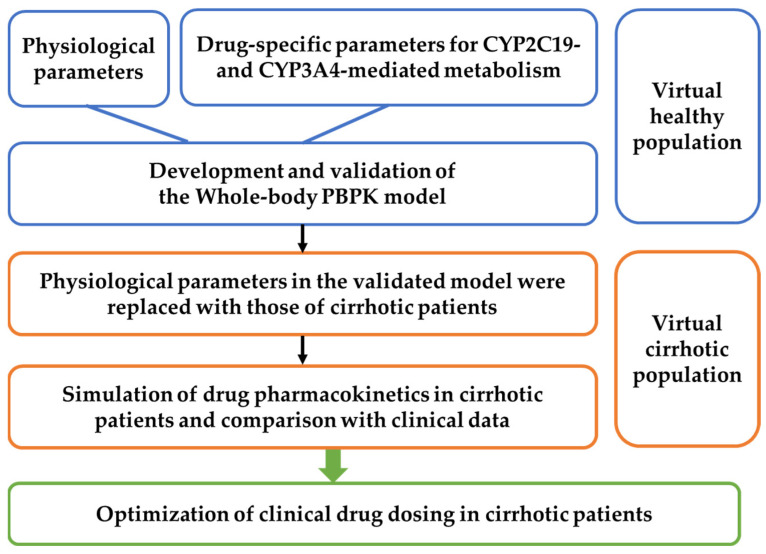
Workflow for developing whole-body PBPK models in healthy individuals and cirrhotic patients and instructing clinical medication.

The workflow for developing the PBPK model applicable to healthy subjects and patients with different grades of cirrhosis is shown in Figure 1. A virtual population–based PBPK model was established to characterize pharmacokinetic variability across individuals with different physiological states. A total of 1000 virtual individuals were generated. Clinically reported mean physiological parameters (Table 1) were used as the typical values. Inter-individual variability was introduced by assigning random effects to key pharmacokinetic determinants, including the effective intestinal permeability coefficient (P_eff_), the blood unbound fraction (f_u_), hepatic metabolic capacity (V_max_ or CL_int_), the blood-to-plasma concentration ratio (R_b_), and the hepatic microsomal protein content (PBSF). These parameters were modeled assuming approximately ±20% variation around their typical values based on clinical and preclinical observations.

To simulate inter-individual and intra-individual variability, an exponential model and an additive residual error model were applied. The first-order conditional estimation with the Lindstrom–Bates method (FOCE L-B) was used during population model fitting. Pharmacokinetic simulations were performed for each virtual individual and repeated 10 times to incorporate stochastic uncertainty associated with random sampling, resulting in a total of 10,000 PK profiles. The simulation results were processed in descriptive statistics in Phoenix (Version 8.4, Certara, Princeton, NJ, USA), and the 5th, 50th, and 95th percentiles were derived to describe the population distribution of PK profiles.

The PBPK model was first developed to simulate the pharmacokinetics of omeprazole and lansoprazole in healthy subjects and validated against clinical pharmacokinetic data. After confirmation of adequate predictive performance in a virtual population of 1000 healthy individuals, the model was adapted to liver cirrhosis by updating system specific physiological and biochemical parameters according to disease severity. Subsequently, pharmacokinetics of omeprazole and lansoprazole were predicted in 1000 virtual individuals representing different cirrhosis grades and compared with published clinical observations. Following successful validation in cirrhotic populations, the established PBPK framework was extended to other drugs primarily metabolized by CYP3A4 and CYP2C19, and dosage optimization for cirrhotic patients was performed using plasma exposure in healthy subjects as the reference for therapeutic equivalence.

### 2.2. Model Development

**Figure 2:**
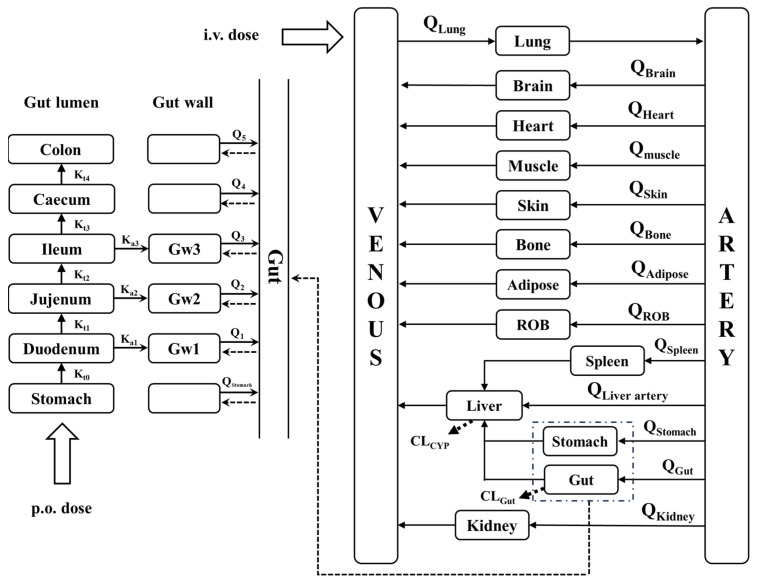
Schematic structure of the whole-body PBPK model. K_ti_ represents the gastric emptying rate and intestinal transit rate. Gwi represents the gut wall of the duodenum, jejunum and ileum. K_ai_ represents the rate of drug absorption into the gut wall. Q represent the blood flow rate. CL represent the clearance. ROB, rest of body (other tissue).

We developed a whole-body PBPK model consisting of intestinal, gut wall, lung, heart, spleen, liver, kidney, brain, adipose, muscle, skin, stomach, arterial blood, venous blood, and the rest of the body (ROB), which are connected by the blood circulatory system (Figure 2).

Drugs are administered either by the intravenous route or the oral route. It is generally accepted that most oral drugs are absorbed in the small intestine (duodenum, jejunum, and ileum) and that the liver is the major metabolizing organ for drugs. In the simulation, it was assumed that the subject drug is eliminated only in the liver and intestine, whereas the absorption of the drug occurs only in the stomach. Drug absorption occurred only in the small intestine. The effective permeability coefficient (P_eff_) was used to indicate the absorption capability of the drug. The essential structure of the whole-body PBPK model and corresponding mass equations were illustrated in Supplementary Information.

### 2.3. PBPK Model Development for LC Patients

Alterations to pharmacokinetics in the liver cirrhosis state are mainly caused by changes in hepatic blood flow, liver volume, enzymes and drug unbound fraction.

The drug unbound fraction for patients with liver cirrhosis (*f_u,cirrhosis_*) are estimated based on a previously published approach [21]. Among these six drugs, except for verapamil and alfentanil, which primarily binds to α1-acid glycoprotein [22,23], the other drugs primarily bind to albumin [24,25,26,27]. According to the pKa values of the drug it was assumed that it binds to either α1-acid glycoprotein or albumin, and the calculation was carried out via Equation (1).

(1) $$\begin{array}{c} f_{u,cirrhosis}=\frac{1}{1+\frac{\left(1-f_{u,healthy}\right)\times C_{prot,cirrhosis}}{C_{prot,healthy}\times f_{u,healthy}}} \end{array}$$

The blood to plasma ratio (*R_B:P_*) was calculated as described before [28]:

(2) $$\begin{array}{c} R_{B:P}=\frac{C_{Blood}}{C_{Plasma}}=\frac{C_{Erythrocyte}}{C_{Plasma}}\times HCT+1-HCT \end{array}$$

*HCT* represents hematocrit (Table 2). The *C_Erythrocyte_*/*C_Plasma_* was calculated from the *R_b_* values reported in healthy individuals and was fixed to zero if the calculated ratio was less than zero.

For the liver volume and blood flow:

(3) $$\begin{array}{c} V_{liver,cirrhosis}=V_{liver}\times \left(Liver\;volume\;fraction\right) \end{array}$$

In the liver, drug metabolism is usually described by the following processes:

(4) $$\begin{array}{c} {CL}_{int,cirrhosis}={CL}_{int}\times \frac{{Enzyme}_{content,cirrhosis}}{{Enzyme}_{content}}\; \end{array}$$

(5) $$\begin{array}{c} V_{max,cirrhosis}=V_{max}\times \frac{{Enzyme}_{content,cirrhosis}}{{Enzyme}_{content}}\; \end{array}$$

The changes in gastric emptying and intestinal peristalsis rate constants were calculated as follows:

(6) $$\begin{array}{c} K_{i,cirrhosis}=K_{i}\times \frac{T_{gastric}}{T_{gastric,cirrhosis}} \end{array}$$

where K_i,cirrhosis_ denotes the gastrointestinal transit rate constant in the cirrhotic state, K_i_ is the gastrointestinal transit rate constant in the normal state, T_gastric,cirrhosis_ denotes the gastrointestinal emptying time in the cirrhotic state, and T_gastric_ denotes the gastrointestinal emptying time in the normal state. The gastric emptying time in liver cirrhosis patients was shortened by 1.26 times, thus K_0_ increased from 0.08 min^−1^ to 0.1 min^−1^. The total gastrointestinal emptying time was shortened from 2 days to 1.6 days. After subtracting the gastric emptying time, the intestinal motility rate increased by approximately 1.25 times, with the values listed in Table 2 [29].

### 2.4. Model Validation

Using the parameters listed in Table 1 and Table 2, plasma concentrations of omeprazole and lansoprazole were predicted in four virtual populations (including normal population, CP-A, CP-B and CP-C patients) following intravenous or oral administration. The drug-specific parameters are listed in Supplementary Tables S1 and S2. The predictions were performed using Phoenix software (Version 8.3.5, Certara, USA, Inc., Princeton, NJ, USA) and were according to the clinical protocol in literatures. The predictions were further compared with clinical observations. To account for inter-individual variability, we used clinically reported mean physiological parameters as a reference and defined variation ranges (e.g., 80–120% of the mean) to represent subject-to-subject differences. By sampling within these ranges, we generated a virtual population of 1000 individuals, each with a unique set of physiological parameters. For each individual, a pharmacokinetic (PK) profile was simulated and replicated 10 times, resulting in a total of 10,000 virtual PK curves. The simulated data were analyzed using the descriptive statistical module in Phoenix, from which the 5th, 50th, and 95th percentile curves were derived to characterize the distribution of PK profiles across the virtual population.

The model was also applied to other drugs with CYP3A4-mediated metabolism (midazolam, ondansetron, verapamil, alfentanil) to simulate the pharmacokinetic process of the drugs in cirrhosis patients and to optimize the dosage of the drugs in the cirrhosis patients.

The PBPK model was deemed successful if the simulated AUC or C_max_ values fell within 0.5- to 2-fold of the observed data or if the observed data lay within the 5th and 95th percentiles of simulations derived from 1000 virtual subjects. At the same time, it also assessed the average fold error (AFE), the average absolute prediction error (PE%) and the geometric mean-fold error (GMFE) of the predictions.

(7) $$\begin{array}{c} AFE=10^{\frac{1}{n}\sum Log\frac{Predi}{Obsi}} \end{array}$$

(8) $$\begin{array}{c} PE\;\%=Geomean \end{array}\left(\left|\frac{Predi-Obsi}{Obsi}\right|\right)\times 100$$

(9) $$\begin{array}{c} GMFE= \end{array}10^{\frac{\sum \left|log(\frac{Predi}{Obsi})\right|}{n}}$$

Prediction results are deemed satisfactory for AFE values between 0.8 and 1.25, acceptable for values between 0.5–0.8 or 1.25–2, and poor for values below 0.5 or above 2. In addition, PE% values within 25% are considered satisfactory, values within 25–50% are acceptable, and values beyond 50% indicate poor prediction performance. GMFE values ≤ 1.25 are regarded as satisfactory, 1.25–2 as acceptable, and >2 as poor [30,31].

**Table 1:** Physiological parameters of healthy individuals used in the PBPK model [32].

Organs	Volume (mL)	Blood Flow (mL/min)
Stomach	160	38
Lungs	1170	5600
Muscle	35,000	750
Heart	310	240
Brain	1450	700
Adipose	10,000	260
Skin	7800	300
Liver	1690	300
Kidneys	280	1240
Spleen	190	80
Rest of body	5100	592
Artery blood	1730	/
Venous blood	3470	/
Duodenum	70	118
Jejunum	209	413
Ileum	139	244
Cecum	116	44
Colon	1116	281
r_1_(cm)	2.00	/
r_2_(cm)	1.63	/
r_3_(cm)	1.45	/
K_0_ (min^−1^)	0.08	/
K_1_ (min^−1^)	0.07	/
K_2_ (min^−1^)	0.03	/
K_3_ (min^−1^)	0.04	/
K_4_ (min^−1^)	0.003	/
K_5_ (min^−1^)	0.001	/

**Table 2:** Physiological parameters of patients with cirrhosis used in the PBPK model.

Parameters	Units	Child–Pugh Class			
		Healthy	CP-A	CP-B	CP-C
Q_total_ [18]	mL/min	5600	6496	7392	7896
Hepatic arterial blood flow [33]	mL/min	300	376	416	509
Liver volume fraction [18]	/	1.0	0.81	0.65	0.53
Functional liver size [28]	/	1	0.91	0.81	0.64
Albumin [18]	g/L	44.7	41.1	33.9	26.3
α1-acid glycoprotein [18]	g/L	0.8	0.57	0.52	0.46
Hematocrit [18]	%	40.9	36.6	32.9	31.9
CYP3A4_liver_ content [34]	pmol/mg protein	137	107	70.2	42.8
CYP3A4_gut_ content [34]	pmol/mg protein	65.4	65.4	39.9	31.7
Duodenal CYP3A4 abundance	nmol	9.7	9.7	5.92	4.70
Jejunal CYP3A4 abundance	nmol	38.4	38.4	23.42	18.62
Ileal CYP3A4 abundance	nmol	22.4	22.4	13.67	10.86
CYP2C19_liver_ content [18]	pmol/mg protein	14	4.50	3.60	1.70
CYP1A2_liver_ content [18]	pmol/mg protein	52	32.9	13.6	6.10
CYP2D6_liver_ content [18]	pmol/mg protein	8.0	6.10	2.60	0.84
PBSF	mg protein	82,472	66,802.32	53,606.8	43,710.16
K_0_	min^−1^	0.08	0.1	0.1	0.1
K_1_	min^−1^	0.07	0.088	0.088	0.088
K_2_	min^−1^	0.03	0.038	0.038	0.038
K_3_	min^−1^	0.04	0.050	0.050	0.050
K_4_	min^−1^	0.003	0.004	0.004	0.004
K_5_	min^−1^	0.001	0.001	0.001	0.001

PBSF is the physiological scale factor, which is calculated based on the Functional liver size ratio. The gastric transit rate constant (K_0–5_) is based on the ratio of gastrointestinal empty time. The CP parameter changes are based on the ratios reported in the literatures and the healthy individual parameters in Table 1.

### 2.5. Sensitivity Analysis

Sensitivity analysis was conducted on key parameters, including gastric emptying rate (K_t_), plasma unbound fraction of the drug (f_u_), and metabolic enzyme activities (characterized by V_max_ or CL_int_ of CYP2C19 and CYP3A4), based on their actual variation ranges.

### 2.6. Dose Optimization

Three virtual patient groups were categorized based on different degrees of cirrhosis, with 1000 individuals in each group. Healthy individuals (70 kg) were used as a reference population. The clinical doses for patients were optimized to ensure that their AUC values were comparable to those of healthy individuals receiving the prescribed dose, with deviations within 15%.

## 3. Results

### 3.1. Drug Data Set

Two drugs primarily metabolized by CYP2C19 and four drugs primarily metabolized by CYP3A4 were selected from data published in PubMed based on the following criteria:

(1) Pharmacokinetic parameters (e.g., AUC or plasma drug concentration) following intravenous and/or oral administration in patients with cirrhosis were available.
(2) Clinical pharmacokinetic data could be obtained from different reports. The collected clinical reports are summarized in Table 3.
(3) The pharmacokinetic data were collected from multiple published studies using different analytical methods, which may introduce variability in the validation dataset and influence the comparison between model predictions and observed values.

### 3.2. CYP2C19 Substrate Drugs

#### Omeprazole and Lansoprazole

**Figure 3:**
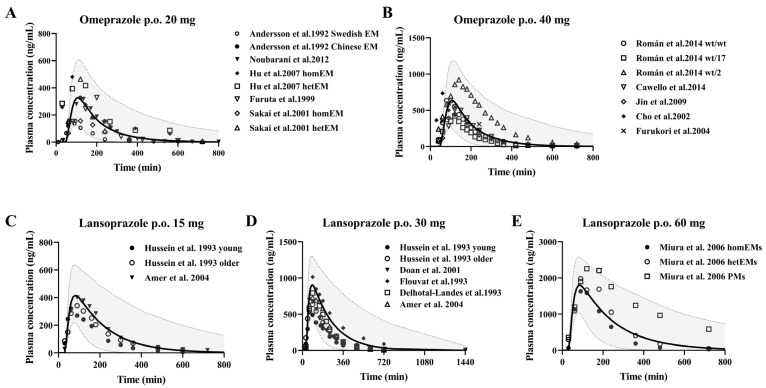
Predicted (line) and observed (dot) plasma concentrations of omeprazole and lansoprazole in healthy subjects. Omeprazole (**A**,**B**) was administered orally at doses of 20 mg [35,36,37,38,39] and 40 mg [40,41,42,43,44] enteric-coated omeprazole capsules; lansoprazole (**C**–**E**) was administered orally at doses of 15 mg [45,46], 30 mg [45,46,47,49,50] and 60 mg [48] enteric-coated omeprazole capsules. The solid line represents the 50th percentile and the dashed lines represent the 5th and 95th percentiles.

After gastric emptying, enteric formulations should dissolve rapidly in the duodenum. Therefore, the administration time from the stomach to the duodenum is defined as the lag time. We assumed that the drug dissolves rapidly when 90% of the drug reaches the duodenum after gastric emptying [70]. These two drugs are metabolized primarily by CYP2C19 to hydroxylated metabolites, and the rest are metabolized by CYP3A4 to sulphonated metabolites in humans. The pharmacokinetic profile of single-dose oral administration of 20 mg and 40 mg omeprazole in healthy individuals, and single-dose oral administration of 10 mg omeprazole in the three cirrhotic states was predicted using the developed PBPK model. Also, the pharmacokinetics of single dose oral administration of 15 mg, 30 mg, 60 mg lansoprazole in healthy individuals and single dose oral administration of 30 mg lansoprazole in cirrhotic states of three grades were predicted. The predictions were then validated with observations from the clinical research literature, respectively, and the predictions are shown in Figure 3.

### 3.3. CYP3A4 Substrate Drugs

#### 3.3.1. Midazolam and Ondansetron

Midazolam is a typical benzodiazepine anaesthetic with anti-anxiety, sedative and hypnotic properties. Midazolam is predominantly metabolized by hepatic CYP3A4, producing approximately 70% 1-hydroxymidazolam and less than 5% 4-hydroxymidazolam. Ondansetron is primarily metabolized to hydroxylated ondansetron by CYP3A4, with additional contributions from CYP2D6 and CYP1A2. The PBPK model was employed to predict the pharmacokinetic profiles of ondansetron administered as a single 8 mg oral dose, single 8 mg intravenous dose, and single 24 mg intravenous dose in a healthy population, as well as a single 8 mg intravenous dose in individuals with three grades of cirrhosis.

#### 3.3.2. Verapamil and Alfentanil

**Figure 4:**
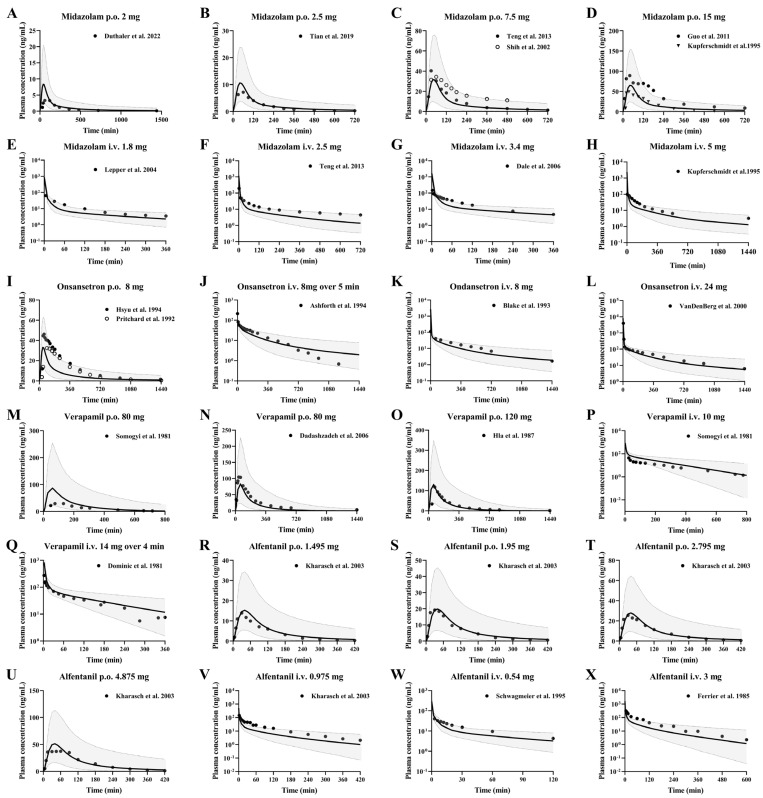
Predicted (line) and observed (dot) plasma concentrations of midazolam, ondansetron, verapamil and alfentanil in healthy subjects. The solid line represents the 50th percentile and the dashed lines represent the 5th and 95th percentiles. Midazolam (**A**–**H**) was administered orally at doses of 2 mg [16], 2.5 mg [51], 7.5 mg [52,54], and 15 mg [53,55], and intravenously at doses of 1.8 mg [56], 2.5 mg [54], 3.4 mg [57], and 5 mg [53]. Ondansetron (**I**–**L**) was administered orally at dose of 8 mg [59,60], infusion at dose of 8 mg over 5 min [61], intravenously at doses of 8 mg [62], 24 mg [58]. Verapamil (**M**–**Q**) was administered orally at doses of 80 mg [65,66], 120 mg [64], intravenously at dose of 10 mg [66], and infusion 14 mg over 4 min [63]. Alfentanil (**R**–**X**) was administered orally at doses of 23 μg/kg (1.495 mg), 30 μg/kg (1.95 mg), 43 μg/kg (2.795 mg), 75 μg/kg (4.875 mg) [67], and intravenously at doses of 15 μg/kg (0.975 mg) [67], 0.54 mg [68] and 3 mg [69].

Verapamil is primarily metabolized in the liver, predominantly by CYP3A4 through N-demethylation to form norverapamil, with additional contributions from CYP3A5 and CYP2C8. Alfentanil is a short-acting opioid analgesic primarily metabolized by the hepatic CYP3A4 enzyme. It also serves as an in vivo probe for assessing hepatic CYP3A4 activity and drug-drug interactions. The developed PBPK model was employed to predict the pharmacokinetics of alfentanil in a healthy population following a single oral dose across four groups, intravenous infusion across three groups, and a single intravenous dose in individuals with cirrhosis under one dosing regimen. The predictions were then validated with observations from the clinical research literature, respectively, and the predictions are shown in Figure 4.

### 3.4. Development of PBPK Model and Validation Using Pharmacokinetic Parameters from Healthy Subjects Following i.v. or Oral Administrations

Plasma concentration-time profiles after intravenous or oral administration of these six drugs in healthy subjects were simulated using the established PBPK model and compared with clinical observations. The AUC or C_max_ values were predicted using 50th percentile profiles and compared with the clinical observations (Table 4, Table 5, Table 6, Table 7, Table 8 and Table 9). 95% (143/150) of the predicted pharmacokinetic parameters were within 0.5–2-fold of the observed values. A few outliers were mainly observed for AUC values. These discrepancies may stem from differences in analytical methods used across studies (such as RIA, HPLC-UV, or LC–MS/MS), which can lead to variability in reported concentrations. In addition, population differences or unmodeled physiological variability might also contribute to these deviations.

### 3.5. Prediction of Pharmacokinetic Profiles of Intravenous or Oral CYP3A4 and CYP2C19 Substrates in Cirrhosis Patients Using the Developed PBPK Model

**Figure 5:**
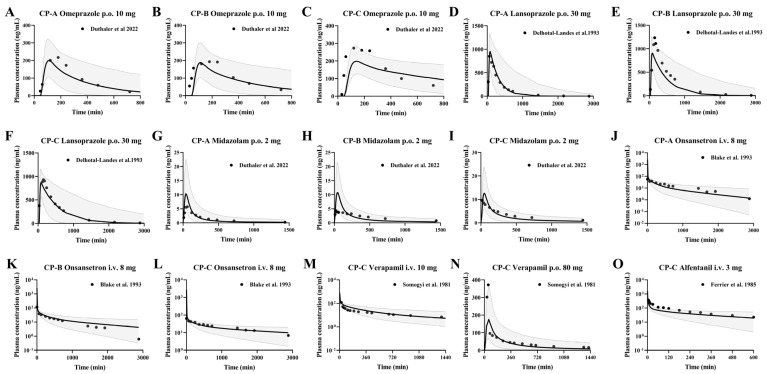
Predicted (line) and observed (dot) plasma concentrations of omeprazole (**A**–**C**), lansoprazole (**D**–**F**), midazolam (**G**–**I**), ondansetron (**J**–**L**), verapamil (**M**,**N**) and alfentanil (**O**) in LC subjects. Omeprazole (**A**−**C**) was administered orally 10 mg enteric-coated omeprazole capsules [16]. Lansoprazole (**D**–**F**) was administered orally 30 mg enteric-coated lansoprazole capsules [50]. Midazolam was administered orally at a dose of 2 mg [16]. Ondansetron was administered intravenously at 8 mg [62]. Verapamil was administered intravenously at 10 mg and orally 80 mg [66]. Alfentanil was administered intravenously at a dose of 3 mg [69]. The solid line represents the 50th percentile and the dashed lines represent the 5th and 95th percentiles.

Following validation of the developed PBPK model in healthy subjects, the model was applied to predict the pharmacokinetic profiles of CYP3A4 and CYP2C19 substrate drugs after intravenous or oral administration in 1000 virtual patients with cirrhosis (Figure 5). Pharmacokinetic parameters were estimated using the mean profiles derived from 1000 simulations. The results indicated that the concentrations of most drugs in cirrhosis patients fell within the 5th and 95th percentiles of the pharmacokinetic profiles of the 1000 virtual cirrhotic patients.

The AFE, PE and GMFE values of six drugs were calculated using parameters of C_max_, T_max_, and AUC_0–t_. The results (Table 10) showed that the predictions for all six drugs were within acceptable ranges.

### 3.6. Sensitivity Analysis of Model Parameters

Plasma concentration-time curves following single-dose oral administration of 20 mg omeprazole, 30 mg lansoprazole, 5 mg midazolam, 80 mg verapamil, and 3 mg alfentanil, as well as intravenous administration of 10 mg verapamil and 8 mg ondansetron, illustrate the sensitivity of pharmacokinetics. Parameters such as gastrointestinal emptying rate, hepatic metabolizing enzyme activity, and plasma unbound drug fraction may influence drug pharmacokinetics. Sensitivity analysis was performed based on the variation ranges of the parameters listed in Table 1 and Table 2. For omeprazole and lansoprazole, sensitivity analyses for CYP2C19 activity were conducted at 1/3-, 1-, and 3-fold to account for the variability in metabolic parameters associated with genetic polymorphisms [70].

**Figure 6:**
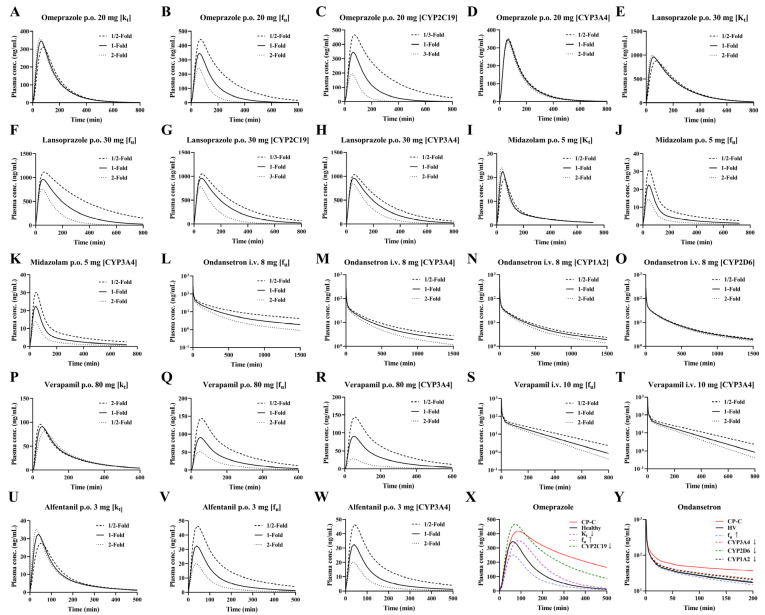
Sensitivity analysis (**A**–**Y**) after oral administration of 20 mg omeprazole (**A**–**D**), 30 mg lansoprazole (**E**–**H**), 5 mg midazolam (**I**–**K**), 80 mg verapamil (**P**), 3 mg alfentanil (**U**–**W**), and intravenous of 8 mg ondansetron (**L**–**O**), 10 mg verapamil (**Q**–**T**). Individual contributions of cirrhosis-induced alterations in K_t_, enzyme activity and f_u_ to plasma concentrations of omeprazole (**X**) and ondansetron (**Y**) following oral 20 mg omeprazole and intravenous of 8 mg ondansetron to LC patients and their integrated effects. HV: healthy volunteer value, CP-C: model predicted value of CP-C with all physiological changes.

The results indicated that the tested parameters affected the pharmacokinetic profiles of the drugs to varying extents (Figure 6). For omeprazole oral, CYP2C19 > f_u_ > K_t_ > CYP3A4; for lansoprazole oral, f_u_ > CYP2C19 > K_t_ >> CYP3A4; for midazolam, verapamil and alfentanil oral, f_u_ ≈ CYP3A4 > K_t_. For ondansetron, f_u_ > CYP3A4 > CYP1A2 > CYP2D6; for verapamil, CYP3A4 > f_u_ > K_t_. For orally administered drugs, the gastric emptying rate (K_t_) primarily influenced the time to peak concentration. CYP2C19 played a more significant role than CYP3A4 in the metabolism of omeprazole and lansoprazole, a 66% reduction in CYP2C19 activity has an effect on AUC that is almost equivalent to a 50% reduction in unbound fraction. Whereas CYP3A4 was the primary contributor for the other four drugs, a 50% reduction in CYP3A4 activity has an effect on AUC that is almost equivalent to a 50% reduction in unbound fraction. Additionally, the plasma unbound drug fraction predominantly affected drug distribution to tissues, making it a key parameter for both intravenous and oral administration routes. The contribution of f_u_, K_t_ and changes in different metabolic enzymes to the plasma concentrations of CYP2C19 and CYP3A4 substrates and their combined effects were also simulated. The results showed that any decrease in metabolizing enzyme activity resulted in an increase in the plasma concentration; whereas a decrease in f_u_ values resulted in a decrease in the plasma concentration. The net effect was an increase in the plasma concentration. For oral drugs (Omeprazole for example), the decrease in the value of K_t_ resulted in delayed absorption of the drug and slowed down the rate of absorption in the intestines, with a significant backward shift of Tmax and an increasing trend in the plasma concentration of the drug.

### 3.7. Dosage Optimization Results

**Figure 7:**
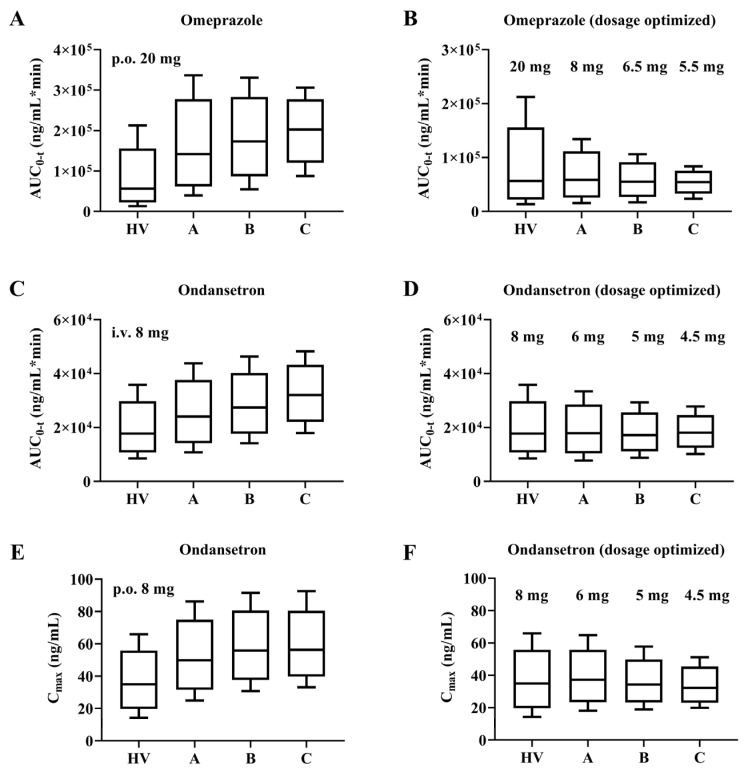
Box-Whisker analysis of omeprazole and ondansetron with adjusted dose design. AUC for omeprazole and ondansetron at clinically recommended doses and after dose optimization (**A**–**D**). Box-Whisker analysis of C_max_ for ondansetron at clinically recommended doses and after adjusted dose design (**E**,**F**).

To illustrate the variability in pharmacokinetics within the cirrhosis patient population, we conducted direct comparisons using box-whisker analysis. As shown in Figure 7, at the clinically prescribed dose administered, the AUC ratio of omeprazole (20 mg orally) in patients with CP-A, CP-B, and CP-C versus healthy subjects was 1:2.5:3.1:3.6; and the AUC ratio of ondansetron (8 mg i.v.) was 1:1.4:1.5:1.8. In addition, for the antiemetic ondansetron, the C_max_ was strongly correlated with the effect; therefore, a C_max_ ratio of 1:1.4:1.6:1.6 was calculated for an oral dose of 8 mg ondansetron.

For plasma exposures to be equivalent, the oral dose of omeprazole should be adjusted to 8 mg (CP-A), 6.5 mg (CP-B), and 5.5 mg (CP-C), while the intravenous dose of ondansetron should be adjusted to 6 mg (CP-A), 5 mg (CP-B), and 4.5 mg (CP-C). When 8 mg ondansetron orally, the dosage should be adjusted to 6 mg (CP-A), 5 mg (CP-B), and 4.5 mg (CP-C). This would suggest that for ondansetron, the dose optimized with AUC and C_max_ as indicators is consistent.

## 4. Discussion

Hepatic drug clearance primarily depends on hepatic blood flow, the activity of drug-metabolizing enzymes, and the concentration of unbound drug in plasma [71,72]. In cirrhosis, histological structural abnormalities lead to changes in hepatic blood flow. As the disease progresses, increased vasodilation contributes to the development of hepatorenal syndrome [73], which subsequently elevates cardiac output [74]. Additionally, altered levels of cholecystokinin in cirrhotic patients impair gallbladder contraction, resulting in prolonged gastric emptying time [75]. In patients with cirrhosis, the overall activity of the CYP450 enzyme system is significantly impaired due to a reduction in hepatocyte number, decreased hepatic blood flow, and the loss of functional liver size. Compared with healthy individuals, CYP2C19 expression is reduced by 32%, 26%, and 12% in CP-A, CP-B, and CP-C patients, respectively, while CYP3A4 expression is reduced by 59%, 39%, and 25% [18]. However, changes in drug exposure cannot be solely determined based on cirrhosis grades, as liver extraction ratio of drugs varies, and the activity of different CYP isoforms is affected to varying degrees by the physiological alterations associated with cirrhosis.

The whole-body PBPK model systematically simulates drug pharmacokinetics in healthy individuals by integrating changes in physiological parameters and extending these simulations to patients with varying degrees of cirrhosis. Compared to empirical PK-PD models or semi-PBPK models, whole-body PBPK models provide more detailed information on physiological parameters and drug metabolism mechanisms, leading to enhanced predictive capability [76,77]. These models facilitate in vivo exposure predictions in the early stages of drug clinical trials, without the need for extensive clinical data [78,79,80]. These predictions offer valuable references for clinical trial dose setting in patients with liver dysfunction. Meanwhile, the construction of a whole-body PBPK model can provide valuable insights into the impact of physiological changes on pharmacokinetics under disease conditions and can be used to optimize drug dosing for patient groups in response to disease progression.

Our simulation results demonstrated that nearly all observed plasma concentrations of the drugs fell within the 5th and 95th percentiles of the virtual population simulations generated by the developed PBPK model. Additionally, the predicted-to-observed ratios for most pharmacokinetic parameters, including AUC and C_max_, were within the range of 0.5 to 2.0, indicating that the whole-body PBPK model accurately predicted the pharmacokinetics of midazolam, omeprazole, lansoprazole, ondansetron, verapamil, and alfentanil in both healthy and cirrhotic populations. Sensitivity analysis was conducted using parameter values reported in the literature and the extent of variation in these parameters in healthy and cirrhotic populations. The results revealed that decreased expression of CYP2C19 or CYP3A4 was the primary contributor to the increased in vivo exposure of their substrate drugs, while changes in gastrointestinal transit rates delayed the time to peak concentration. Moreover, elevated plasma unbound drug fractions led to greater drug distribution into tissues, reducing plasma concentrations transiently; however, the net effect was an increase in plasma drug exposure [81].

It is widely acknowledged that dose adjustments are necessary for cirrhotic patients when drug exposure levels exceed twice that of the healthy population. According to our model predictions, the AUC ratios of omeprazole (20 mg orally) in patients with CP-A, CP-B, and CP-C compared to healthy subjects were 1:2.5:3.1:3.6, respectively. In contrast, the AUC ratios of ondansetron (8 mg intravenously) were 1:1.4:1.5:1.8. These findings highlight that, even within the same cirrhosis grade, some drugs may not require additional dose adjustments, whereas others could lead to significant drug accumulation. Although it is reasonable to use AUC and C_max_ as indicators for dose optimization, for more extensive situations, it may be necessary to consider indicators such as drug exposure in specific organs or the duration of exposure above threshold concentrations to ensure drug safety. This underscores the potential utility of the PBPK model for optimizing clinical dosing in cirrhotic patients.

However, this study has certain limitations. Due to the limited availability of human physiological data and corresponding clinical studies in cirrhotic patients, the PBPK model primarily accounted for changes in parameters such as hepatic blood flow, CYP450 enzyme activity, cardiac output, and plasma unbound drug fraction. Other potential influencing factors, such as the impact of cirrhosis on metabolizing enzyme activity across different ages or genotypes, were not included [82]. It was demonstrated that for the CYP2C9 substrate drug, lornoxicam, the effect of cirrhosis grade on pharmacokinetics was greater than the effect of genotypic differences [83]. And this study focused on the effect of CYP2C19 and CYP3A4 impairment on drug exposure, the model structure already includes dynamic enzyme activity and substrate-competition terms, indicating its potential for drug–drug interaction assessment when reliable inhibition/induction parameters are available. Future extensions of the model may therefore support optimisation of combination therapy in cirrhotic patients. Although the complexity of the discussion limits genotypic depth, our model provided a basis for accurate assessment of genotypes for CYP2C19 and CYP3A4 substrate drugs subsequent studies. Additionally, although the Child-Pugh class was used as a category in our study to simulate pharmacokinetic changes in different hepatic function states, it is important to note that this system of comprehensive categories based on a wide range of clinical physiological parameters does not adequately reflect the variability of physiological parameters of the patients in each class. The PBPK model was developed using typical parameter values and an assumed 20% variation, which may underestimate the inter-individual pharmacokinetic variability observed in real populations. Future refinements could incorporate known genetic variability to improve prediction accuracy. In future applications expansion, the individual variability values of real patients could be integrated to improve prediction accuracy and clinical adaptability. In data collection, we included as many relevant publications as possible. However, different detection methods vary in terms of sensitivity, specificity, and interference resistance. For example, RIA methods may have cross-reactivity or poor specificity, which may affect the accuracy of some blood drug concentration data.

## 5. Conclusions

A whole-body PBPK model of CYP2C19 and CYP3A4 substrates containing omeprazole, lansoprazole, midazolam, ondansetron, verapamil, and alfentanil was developed in healthy subjects. After validation with clinical data reported in literatures, the PBPK model was applied to patients with different grades of cirrhosis to predict pharmacokinetic profiles. The developed PBPK model can also be used to guide dose optimization in the liver cirrhosis patient population.

## Figures and Tables

**Table 3:** Clinical information about CYP2C19 and CYP3A4 substrates in the simulations.

No	Research	Drug	Dose	Assays	Subjects (n)	Refs.
1	Andersson et al., 1992	Omeprazole	p.o. 20 mg	HPLC	Healthy (14)	[35]
2	Noubarani et al., 2012	Omeprazole	p.o. 20 mg	LC-MS/MS	Healthy (30)	[36]
3	Hu et al., 2007	Omeprazole	p.o. 20 mg	HPLC-UV	Healthy (18)	[37]
4	Sakai et al., 2001	Omeprazole	p.o. 20 mg	HPLC-UV	Healthy (18)	[38]
5	Furuta et al., 1999	Omeprazole	p.o. 20 mg	HPLC-UV	Healthy (6)	[39]
6	Román et al., 2014	Omeprazole	p.o. 40 mg	LC-MS	Healthy (35)	[40]
7	Cawello et al., 2014	Omeprazole	p.o. 40 mg	LC-MS	Healthy (36)	[41]
8	Jin et al., 2009	Omeprazole	p.o. 40 mg	HPLC-UV	Healthy (9)	[42]
9	Cho et al., 2002	Omeprazole	p.o. 40 mg	HPLC-UV	Healthy (8)	[43]
10	Furukori et al., 2004	Omeprazole	p.o. 40 mg	HPLC-UV	Healthy (6)	[44]
11	Duthaler et al., 2022	Omeprazole	p.o. 10 mg	LC-MS/MS	CP (A-C) (36)	[16]
12	Hussein et al., 1993	Lansoprazole	p.o. 15; 30 mg	HPLC-UV	Healthy (24)	[45]
13	Amer et al., 2004	Lansoprazole	p.o. 15; 30 mg	LC-MS	Healthy (36)	[46]
14	Doan et al., 2001	Lansoprazole	p.o. 30 mg	LC-MS	Healthy (36)	[47]
15	Miura et al., 2006	Lansoprazole	p.o. 60 mg	HPLC	Healthy (18)	[48]
16	Flouvat et al., 1993	Lansoprazole	p.o. 30 mg	HPLC-	Healthy (18)	[49]
17	Delhotal-Landes et al., 1993	Lansoprazole	p.o. 30 mg	HPLC	CP (A-C) (24)	[50]
18	Tian et al., 2019	Midazolam	p.o. 2.5 mg	LC-MS/MS	Healthy (8)	[51]
19	Shih et al., 2002	Midazolam	p.o. 7.5 mg	HPLC-UV	Healthy (42)	[52]
20	Kupferschmidt et al., 1995	Midazolam	p.o. 15 mg	HPLC-UV	Healthy (8)	[53]
21	Teng et al., 2013	Midazolam	p.o. 7.5 mg; i.v. 2.5 mg	LC-MS/MS	Healthy (28)	[54]
22	Guo et al., 2011	Midazolam	p.o. 15 mg	HPLC	Healthy (49)	[55]
23	Lepper et al., 2004	Midazolam	i.v. 1.8 mg	LC-MS	Healthy (35)	[56]
24	Dale et al., 2006	Midazolam	i.v. 3.4 mg	GC-MS	Healthy (12)	[57]
25	Duthaler et al., 2022	Midazolam	p.o. 2 mg	LC-MS/MS	CP (A-C) (36)	[16]
26	VanDenBerg et al., 2000	Ondansetron	p.o. 24 mg	HPLC-UV	Healthy (12)	[58]
27	Hsyu et al., 1994	Ondansetron	p.o. 8 mg	HPLC-UV	Healthy (6)	[59]
28	Pritchard et al., 1992	Ondansetron	p.o. 8 mg	HPLC-UV	Healthy (11)	[60]
29	Ashforth et al., 1994	Ondansetron	i.v. 8 mg over 5 min	GC-UV	Healthy (12)	[61]
30	Blake et al., 1993	Ondansetron	i.v. 8 mg over 5 min	HPLC	CP (A-C) (19)	[62]
31	Dominic et al., 1981	Verapamil	i.v. 14 mg over 4 min	GC-UV	Healthy (8)	[63]
32	Hla et al., 1987	Verapamil	p.o. 120 mg	HPLC	Healthy (10)	[64]
33	Dadashzadeh et al., 2006	Verapamil	p.o. 80 mg	HPLC-FLD	Healthy (12)	[65]
34	Somogyi et al., 1981	Verapamil	p.o. 80 mg i.v. 10 mg	LC-UV	Healthy (6) CP (C) (7)	[66]
35	Kharasch et al., 2003	Alfentanil	i.v. 0.975 mg; p.o. 1.495 mg; 1.95 mg; 2.795 mg; 4.875 mg	LC-MS	Healthy (10)	[67]
36	Schwagmeier et al., 1995	Alfentanil	i.v. 0.54 mg	RIA	Healthy (10)	[68]
37	Ferrier et al., 1985	Alfentanil	i.v. 3 mg	RIA	Healthy (10) CP (C) (11)	[69]

**Table 4:** Observed and predicted values of C_max_, T_max_ and AUC_0–t_ of omeprazole in healthy subjects and liver cirrhotic patients.

Dose	Subjects	C_max_ (ng/mL)			T_max_ (Hour)			AUC_0–t_ (ng × Hour/mL)		
		Obs	Pre	Ratio	Obs	Pre	Ratio	Obs	Pre	Ratio
20 mg [35]	Healthy	258.37 ± 99.48	329.75	1.28	NR	/	/	324.69 ± 120.9	926.13	2.85 *
20 mg [35]	Healthy	203.11 ± 81.17	329.75	1.62	NR	/	/	905.00 ± 614.85	926.13	1.02
20 mg [36]	Healthy	397.2	329.75	0.83	1.9	2.3	1.21	825.1	939.37	1.14
20 mg [37]	Healthy	513.9 ± 294.8	329.75	0.64	NR	/	/	1644.6 ± 745.8	920.16	0.56
20 mg [37]	Healthy	566.8 ± 294.8	329.75	0.58	NR	/	/	1759.4 ± 838.6	920.16	0.52
20 mg [38]	Healthy	251.2 ± 46.2	329.75	1.31	2.8 ± 0.3	2.3	0.82	623.1 ± 149.1	936.21	1.50
20 mg [38]	Healthy	618.3 ± 141.9	329.75	0.53	3.0 ± 0.6	2.3	0.77	1061.8 ± 269.2	936.21	0.88
20 mg [39]	Healthy	NR	/	/	NR	/	/	383.9 ± 26.3	926.13	2.41 *
20 mg [39]	Healthy	NR	/	/	NR	/	/	1001.9 ± 160.3	926.13	0.92
40 mg [40]	Healthy	834.37 ± 394.54	634.89	0.76	NR	/	/	1658.69 ± 1271.58	1749.81	1.05
40 mg [40]	Healthy	1266.48 ± 477.05	634.89	0.50	NR	/	/	3887.72 ± 2087.92	1749.81	0.45 *
40 mg [40]	Healthy	722.76 ± 346.14	634.89	0.88	NR	/	/	1096.7 ± 646.53	1749.81	1.60
40 mg [41]	Healthy	586	634.89	1.08	2	2.3	1.15	1027	1478.09	1.44
40 mg [42]	Healthy	723.0 ± 67.3	634.89	0.88	2.1 ± 0.2	2.3	1.10	1385.6 ± 183.7	1733.32	1.25
40 mg [43]	Healthy	986.56	634.89	0.64	1.83	2.3	1.26	1834.34	1834.34	1.00
40 mg [44]	Healthy	900	634.89	0.71	1.75	2.3	1.31	1481	1691.35	1.14
10 mg [16]	CP-A	218	188.3	0.86	NR	/	/	986	1015.91	1.03
10 mg [16]	CP-B	273	198.67	0.73	NR	/	/	1327	1123.19	0.85
10 mg [16]	CP-C	343	199.34	0.58	NR	/	/	2111	1676.53	0.79

NR: Not reported. * Indicates more than 0.5–2-fold range.

**Table 5:** Observed and predicted values of C_max_, T_max_ and AUC_0–t_ of lansoprazole in healthy subjects and liver cirrhotic patients.

Dose	Subjects	C_max_ (ng/mL)			T_max_ (Hour)			AUC_0–t_ (ng × Hour/mL)		
		Obs	Pre	Ratio	Obs	Pre	Ratio	Obs	Pre	Ratio
15 mg [45]	Healthy	413 ± 199	415.06	1.00	1.15 ± 0.39	1.5	1.30	950 ± 593	1398.42	1.47
15 mg [45]	Healthy	449 ± 150	415.06	0.92	1.46 ± 0.53	1.5	1.03	1334 ± 673	1398.42	1.05
15 mg [46]	Healthy	578.6	415.06	0.72	1.7	1.5	0.88	1451	1421.86	0.98
30 mg [45]	Healthy	750 ± 331	902.90	1.20	1.48 ± 0.99	1.5	1.01	1763 ± 1056	3449.40	1.96
30 mg [45]	Healthy	773 ± 248	902.90	1.17	1.56 ± 0.94	1.5	0.96	2678 ± 1144	3449.40	1.29
30 mg [46]	Healthy	1077	902.90	0.84	1.8	1.5	0.83	2641	3546.69	1.34
30 mg [47]	Healthy	810.4 ± 340.5	902.90	1.11	1.6 ± 0.7	1.5	0.94	2157 ± 1933	3709.69	1.72
30 mg [49]	Healthy	1148 ± 536	902.90	0.79	1.5	1.5	1.00	5216 ± 3855	3709.69	0.71
30 mg [50]	Healthy	1033	902.90	0.87	1.5	1.5	1.00	2670	3546.69	1.33
60 mg [48]	Healthy	1957 ± 413	1801.38	0.92	1.9 ± 0.6	1.5	0.79	5009 ± 919	6865.35	1.37
60 mg [48]	Healthy	2196 ± 405	1801.38	0.82	2.3 ± 0.8	1.5	0.65	7300 ± 1008	6865.35	0.94
60 mg [48]	Healthy	2516 ± 357	1801.38	0.72	2.4 ± 0.9	1.5	0.63	20,132 ± 3570	6865.35	0.34 *
30 mg [50]	CP-A	1080	864.29	0.80	1.4	1.5	1.07	5200	4841.99	0.93
30 mg [50]	CP-B	1440	904.69	0.63	2.1	1.5	0.71	11,700	7453.58	0.64
30 mg [50]	CP-C	1140	951.89	0.83	2.1	1.5	0.71	10,700	8305.20	0.78

NR: Not reported. * Indicates more than 0.5–2-fold range.

**Table 6:** Observed and predicted values of C_max_, T_max_ and AUC_0–t_ of midazolam in healthy subjects and liver cirrhotic patients.

Dose	Subjects	C_max_ (ng/mL)			T_max_ (Hour)			AUC_0–t_ (ng × Hour/mL)		
		Obs	Pre	Ratio	Obs	Pre	Ratio	Obs	Pre	Ratio
p.o. 2.5 mg [51]	Healthy	7.56	10.62	1.40	1	0.67	0.67	20.69	26.80	1.30
p.o. 2 mg [16]	Healthy	4.44	8.33	1.88	NR	/	/	11.9	25.06	2.11 *
p.o. 7.5 mg [52]	Healthy	NR	/		NR	/	/	153.95 ± 17.5	81.87	0.53
p.o. 7.5 mg [54]	Healthy	47.3	31.61	0.67	0.75	0.67	0.89	115.4	81.87	0.71
p.o. 15 mg [53]	Healthy	54.3 ± 6.4	65.08	1.20	0.62 ± 0.20	0.67	1.08	143 ± 26	157.84	1.10
p.o. 15 mg [55]	Healthy	116.2 ± 61.0	65.08	0.56	1.0 ± 0.06	0.67	0.67	330.7 ± 139.6	195.93	0.59
p.o. 15 mg [55]	Healthy	130.2 ± 59.4	65.08	0.50	1.0 ± 0.8	0.67	0.67	365.0 ± 103.8	195.93	0.54
i.v. 1.8 mg [56]	Healthy	NR	/	/	NR	/	/	96.1 ± 42.7	109.00	1.13
i.v. 2.5 mg [54]	Healthy	NR	/	/	NR	/	/	115.7	161.33	1.39
i.v. 3.4 mg [57]	Healthy	NR	/	/	NR	/	/	117.03	208.16	1.78
i.v. 5 mg [53]	Healthy	NR	/	/	NR	/	/	199	351.01	1.76
p.o. 2 mg [16]	CP-A	6.37	10.32	1.62	NR	/	/	20	28.95	1.45
p.o. 2 mg [16]	CP-B	5.83	10.73	1.84	NR	/	/	29.7	38.49	1.30
p.o. 2 mg [16]	CP-C	11.2	12.62	1.13	NR	/	/	58.1	55.42	0.95

NR: Not reported. * Indicates more than 0.5–2-fold range.

**Table 7:** Observed and predicted values of C_max_, T_max_ and AUC_0–t_ of ondansetron in healthy subjects and liver cirrhotic patients.

Dose	Subjects	C_max_ (ng/mL)			T_max_ (Hour)			AUC_0–t_ (ng × Hour/mL)		
		Obs	Pre	Ratio	Obs	Pre	Ratio	Obs	Pre	Ratio
i.v. 24 mg [58]	Healthy	NR	/	/	NR	/	/	1276.4 ± 340.4	1650.83	1.29
p.o. 8 mg [59]	Healthy	40 ± 22	33.05	0.83	1.3 ± 0.7	0.67	0.52	225 ± 79	123.83	0.55
p.o. 8 mg	Healthy	NR	/	/	NR	/	/	NR	/	/
i.v. 8 mg over 5 min [61]	Healthy	NR	/	/	NR	/	/	257	199.67	0.78
i.v. 8 mg over 5 min [61]	/	/	/	/	/	/	/	247	199.67	0.81
i.v. 8 mg [62]	Healthy	NR	/	/	NR	/	/	279	536.51	1.92
i.v. 8 mg [62]	CP-A	NR	/	/	NR	/	/	633	736.64	1.16
i.v. 8 mg [62]	CP-B	NR	/	/	NR	/	/	446	940.72	2.11 *
i.v. 8 mg [62]	CP-C	NR	/	/	NR	/	/	1383	1226.70	0.89

NR: Not reported. * Indicates more than 0.5–2-fold range.

**Table 8:** Observed and predicted values of C_max_, T_max_ and AUC_0–t_ of verapamil in healthy subjects and liver cirrhotic patients.

Dose	Subjects	C_max_ (ng/mL)			T_max_ (Hour)			AUC_0–t_ (ng × Hour/mL)		
		Obs	Pre	Ratio	Obs	Pre	Ratio	Obs	Pre	Ratio
p.o. 120 mg [64]	Healthy	123 ± 43	129.83	1.06	1.05 ± 0.28	1	0.95	450 ± 130	412.53	0.92
p.o. 80 mg [65]	Healthy	130.66 ± 33.58	82.89	0.63	0.54 ± 0.18	1	1.85	383.67 ± 110.54	261.50	0.68
p.o. 80 mg [65]	Healthy	139.28 ± 77.88	82.89	0.60	0.69 ± 0.24	1	1.45	344.22 ± 239.90	261.50	0.76
p.o. 80 mg [66]	Healthy	38.6	82.89	0.47	NR	/	/	NR	/	/
i.v. 14 mg over 4 min [63]	Healthy	NR	/	/	NR	/	/	NR	/	/
p.o. 80 mg [66]	CP-C	134.5	175.14	1.30	NR	/	/	NR	/	/
i.v. 10 mg [66]	CP-C	NR	/	/	NR	/	/	NR	/	/

NR: Not reported.

**Table 9:** Observed and predicted values of C_max_, T_max_ and AUC_0–t_ of alfentanil in healthy subjects and liver cirrhotic patients.

Dose	Subjects	C_max_ (ng/mL)			T_max_ (Hour)			AUC_0–t_ (ng × Hour/mL)		
		Obs	Pre	Ratio	Obs	Pre	Ratio	Obs	Pre	Ratio
p.o. 1.495 mg [67]	Healthy	16 ± 8	15.29	0.96	0.6 ± 0.3	0.67	1.12	26 ± 14	32.31	1.24
p.o. 1.95 mg [67]	Healthy	23 ± 16	19.80	0.86	0.7 ± 0.4	0.67	0.96	38 ± 22	41.90	1.10
p.o. 2.795 mg [67]	Healthy	31 ± 18	27.83	0.90	0.9 ± 0.8	0.67	0.74	57 ± 31	57.87	1.02
p.o. 4.895 mg [67]	Healthy	50 ± 22	51.45	1.03	0.7 ± 0.5	0.67	0.96	105 ± 59	107.40	1.02
i.v. 0.975 mg [67]	Healthy	NR	/	/	NR	/	/	NR	/	/
i.v. 0.54 mg [68]	Healthy	NR	/	/	NR	/	/	33.10 ± 13.75	21.94	0.66
i.v. 3 mg [69]	Healthy	NR	/	/	NR	/	/	NR	/	/
i.v. 3 mg [69]	CP-C	NR	/	/	NR	/	/	NR	/	/

NR: Not reported.

**Table 10:** Prediction performance of the six drugs in healthy and cirrhotic subjects.

Drugs	Values	C_max_	T_max_	AUC_0–t_
Omeprazole	AFE	0.80	1.07	1.06
	PE	26.47%	19.34%	22.48%
	GMFE	1.41	1.22	1.40
Lansoprazole	AFE	0.88	0.88	1.04
	PE	13.50%	11.79%	22.84%
	GMFE	1.21	1.19	1.40
Midazolam	AFE	1.08	0.78	1.09
	PE	41.17%	20.63%	32.81%
	GMFE	1.57	1.32	1.48
Ondansetron	AFE	0.83	0.52	1.08
	PE	17.38%	48.46%	31.88%
	GMFE	1.21	1.94	1.45
Verapamil	AFE	0.75	1.37	0.78
	PE	30.97%	26.32%	18.54%
	GMFE	1.51	1.41	1.28
Alfentanil	AFE	0.93	0.93	0.99
	PE	6.54%	8.60%	7.82%
	GMFE	1.09	1.13	1.17

Absolute Fold Error (AFE), Prediction Error (PE), and Geometric Mean Fold Error (GMFE).

## Data Availability

Data are contained within the article and the Supplementary Materials.

## Supplementary Materials

The following supporting information can be downloaded at: https://www.mdpi.com/article/10.3390/pharmaceutics17121582/s1, K_t:p_ values were calculated using the method of Rodgers et al [84]. Table S1. Drug-specific parameters primarily mediated by CYP2C19 metabolism [70,85,86,87,88,89,90,91,92]; Table S2. Drug-specific parameters primarily mediated by CYP3A4 metabolism [93,94,95,96,97,98,99,100,101].

## Abbreviations

The following abbreviations are used in this manuscript:

AUC	the area under the curve
AUC_0–t_	the area under the curve from 0 to the last measurable concentration
C_max_	the peak concentration
T_max_	time to peak drug concentration
CYP2C19	Cytochrome P450 2C19
CYP3A4	Cytochrome P450 3A4
LC	liver cirrhosis
f_u_	the unbound fraction of drug
CL	clearance
R_b_	the ratio of drug concentration in blood to plasma
PBSF	the physiologically based scaling factor
K_t_	the transit rate constant
K_t:p_	the ratio of drug concentration in tissue to plasma
PBPK	physiologically based pharmacokinetic
