# Supplementary material for: Pharmacokinetics of CYP2C19- and CYP3A4-Metabolized Drugs in Cirrhosis Using a Whole-Body PBPK Approach
Source: Pharmaceutics. 2025 Dec 8;17(12):1582. doi: 10.3390/pharmaceutics17121582 (PMC12737180; doi:10.3390/pharmaceutics17121582)
Supplement: Supplementary file 1 [file pharmaceutics-17-01582-s001.zip › pharmaceutics-3983022-supplementary.pdf]

Supplementary Materials: Pharmacokinetics of CYP2C19- and CYP3A4-Metabolized Drugs in Cirrhosis Using a Whole-Body PBPK Approach

Ruijing Mu, Jingjing Gao, Xiaoli Wang, Jing Ling, Nan Hu and Hanyu Yang

S1. Whole-body PBPK model development

The developed PBPK model consists of 12 compartments, including arterial blood, venous blood, lung, brain, heart, kidneys, skin, muscle, adipose, liver, intestines, spleen, and rest of the body.

In general tissue compartments (t):

$$dA_{\mathrm{t}}/d\mathrm{t} = Q_{\mathrm{t}} \times \left(C_{\mathrm{A}} - \frac{A_{\mathrm{t}}/V_{\mathrm{t}}}{K_{\mathrm{t:p}}/R_{\mathrm{b}}}\right) \quad \text{(S1)}$$

Where V_t, A_t, Q_t and $K_{t:p}$ represent the volume of tissue or organ, drug amount, blood flow rate, and ratio of drug concentration in tissue to plasma, respectively. C_A is drug concentrations in arterial blood. R_b represents ratio of drug concentration in blood to plasma. $K_{t:p}$ values were calculated using the method of Rodgers et al[1].

It is assumed that drugs are completely absorbed from the intestine. The absorption of drugs occurs in the duodenum, jejunum, and ileum.

In the stomach:

$$dA_0/d\mathrm{t} = -K_{\mathrm{t0}} \times A_0 \quad \text{(S2)}$$

Due to the instability of omeprazole and lansoprazole in the acidic environment of the stomach, these drugs are administered as enteric-coated formulations in clinical practice. To ensure stability in the stomach and enable absorption in the intestine, the enteric coating must remain intact under acidic conditions. Once gastric emptying occurs, the drug is expected to dissolve rapidly in the duodenum. Consequently, the time required for the drug to transit from the stomach to the duodenum is referred to as the lag time.[2]

In the gut lumen, for normal oral solutions or immediate-release drugs (A_i):

$$dA_{\mathrm{i}}/d\mathrm{t} = K_{\mathrm{ti-1}} \times A_{\mathrm{i-1}} - K_{\mathrm{ti}} \times A_{\mathrm{i}} - k_{\mathrm{ai}} \times A_{\mathrm{i}} \quad \text{(S3)}$$

Where i=1, 2, 3 indicate duodenum, jejunum and ileum, respectively. K_{ti} represents constant of intestinal transit, K_{ai} is constant of absorption rate, which could be estimated using the equations.

$$K_{\mathrm{ai}} = \frac{2 \times P_{\mathrm{eff}}}{r_{\mathrm{i}}} \quad \text{(S4)}$$

$$\log P_{\mathrm{eff}} = 0.4926 \times \log P_{\mathrm{app},caco-2} - 0.1454 \quad \text{(S5)}$$

Where P_{eff} and r_i are human effective permeability and radius of the gut lumen.

In the gut wall (A_{Gwi}):

$$dA_{\mathrm{Gwi}}/d\mathrm{t} = Q_{\mathrm{Gwi}} \times \left(C_{\mathrm{A}} - \frac{A_{\mathrm{G}wi}/V_{\mathrm{G}wi}}{K_{\mathrm{g:p}}/R_{\mathrm{b}}}\right) + k_{\mathrm{ai}} \times A_{\mathrm{i}} \quad \text{(S6)}$$

Where V_{Gwi}, Q_{Gwi} and $K_{g:p}$ represent the volume of intestinal wall, blood flow rate and ratio of drug concentrations in intestine to plasma, respectively.

In the liver compartment:

$$dA_{\mathrm{Liv}}/dt = Q_{\mathrm{La}} \times C_{\mathrm{A}} + \Sigma Q_{\mathrm{Gwi}} \times \frac{A_{\mathrm{Gw}i}/V_{\mathrm{Gw}i}}{K_{\mathrm{g:p}}/R_{\mathrm{b}}} + Q_{\mathrm{Sp}} \times \frac{A_{\mathrm{Sp}}/V_{sp}}{K_{\mathrm{Sp:p}}/R_{\mathrm{b}}} - (Q_{\mathrm{Liv}} + CL_{liver}) \times \frac{A_{Liv}/V_{Liv}}{K_{\mathrm{Liv:}p}/R_{\mathrm{b}}} \quad \text{(S7)}$$

Q_{La} and Q_{liv} are the blood flow rate of the hepatic artery and liver blood flow, respectively, where $Q_{Liv}=Q_{La}+Q_{sp}+\sum Q_{Gwi}$. Sp indicates spleen. CL_{liver} is the clearance rate from the liver. The rate of drug clearance was described using the Michaelis-Menten equation:

$$CL_{liver} = \frac{V_{max} \times PBSF(\times Enzyme\ content)}{K_m + f_u \times \frac{A_{Liv}/V_{Liv}}{K_{\mathrm{Liv:}p}/R_{\mathrm{b}}}} \quad \text{(S8)}$$

V_{max} represents the maximum enzyme-mediated metabolic rate, with units of pmol/min/mg protein, corrected using PBSF. When the units are pmol/min/pmol CYP, further correction is performed using enzyme content. It is assumed that the degree of reduction in the expression of metabolic enzymes in the liver represents the degree of functional reduction.

In venous blood compartment (V):

$$dA_{\mathrm{v}}/d\mathrm{t} = \Sigma\left(Q_{\mathrm{t}} \times \frac{C_{\mathrm{t}}}{K_{\mathrm{t:p}}/R_{\mathrm{b}}}\right) - Q_{\mathrm{total}} \times A_{\mathrm{v}}/V_{\mathrm{v}} \quad \text{(S9)}$$

Where Q_{total} represents cardiac output.

In the artery (A) and lung (Lu) compartments:

$$dA_{\mathrm{A}}/d\mathrm{t} = Q_{\mathrm{total}} \times \left(\frac{A_{\mathrm{Lu}}/V_{\mathrm{Lu}}}{K_{\mathrm{Lu:p}}/R_{\mathrm{b}}} - A_{\mathrm{A}}/V_{\mathrm{A}}\right) \quad \text{(S10)}$$

$$dA_{\mathrm{Lu}}/d\mathrm{t} = Q_{\mathrm{total}} \times \left(A_{\mathrm{v}}/V_{\mathrm{v}} - \frac{A_{\mathrm{Lu}}/V_{\mathrm{Lu}}}{K_{\mathrm{Lu:p}}/R_{\mathrm{b}}}\right) \quad \text{(S11)}$$

V_A and V_V are volume of arterial blood and venous blood, respectively.

Table S1. Drug-specific parameters primarily mediated by CYP2C19 metabolism.

Parameters	Units	Omeprazole	Lansoprazole
MW [a]	g/mol	345.4	369.4
logP [a]	/	1.66	2.1[3]
pKa [a]	/	4.77	8.5[3]
R_b	/	0.59[4]	0.74[5]
f_u	/	0.03[2]	0.075[6]
P_{app}	10^{-4} cm/s	16.8[2]	7.15[5]
$K_{m,2C19}$	μM	1.46[7]	16.8[8]
$V_{max,2C19}$	pmol/min/pmol CYP	15.05[9]	/
$V_{max,2C19}$	pmol/min/mg protein	/	318.8[8]
$K_{m,3A4,\ sul}$	μM	140[10]	136.2[8]
$V_{max,3A4,\ sul}$	nmol/min/nmol CYP	13[10]	/
$V_{max,3A4,\ sul}$	pmol/min/mg protein	/	1387.5[8]
$K_{m,3A4,\ hydroxy}$	μM	60[10]	/
$V_{max,3A4,\ hydroxy}$	nmol/min/nmol CYP	2.4[10]	/

[a] From DrugBank (https://www.drugbank.ca/). pKa, acid dissociation constant; logP, the log of the partition coefficient of a solute between octanol and water; f_u, fraction unbound in plasma; R_b, blood to plasma ratio; Papp, permeability coefficient.

Table S2. Drug-specific parameters primarily mediated by CYP3A4 metabolism.

Parameters	Units	Midazolam	Ondansetron	Verapamil	Alfentanil
MW [a]	g/mol	325.8	293.4	454.6	416.5
logP [a]	/	2.75	2.56	2.84[11]	2.1
pKa [a]	/	6.19	7.34	9.68	7.5
R_b	/	0.60[12]	0.85[13]	0.76[12]	0.55[14]
f_u	/	0.032[15]	0.27[16]	0.13[12]	0.086[17]
P_{app}	10^{-4} cm/s	2.03[18]	4.17[12]	1.3[12]	3.9[12]
$K_{m, 1\text{-}OH\text{-}M}$	μM	2.18[20]	/	/	/
$V_{max, 1\text{-}OH\text{-}M}$	pmol/min/pmol CYP	5.23[20]	/	/	/
$K_{m, 4\text{-}OH\text{-}M}$	μM	31.8[20]	/	/	/
$V_{max,4\text{-}OH\text{-}M}$	pmol/min/pmol CYP	5.2[20]	/	/	/
$CL_{int,3A4}$	μL/min/pmol CYP	/	0.13[13]	/	/
$CL_{int,3A4}$	μL/min/mg protein	/	/	262[12]	135[17]
$CL_{int,1A2}$	μL/min/pmol CYP	/	0.24[13]	/	/
$CL_{int,2D6}$	μL/min/pmol CYP	/	0.54[13]	/	/

[a] From DrugBank (https://www.drugbank.ca/).

References

[1]. Rodgers, T.; Leahy, D.;Rowland, M., Physiologically based pharmacokinetic modeling 1: predicting the tissue distribution of moderate-to-strong bases. *J Pharm Sci* **2005,** *94*, 1259-76.

[2]. Li, S.; Xie, L.; Yang, L.; Jiang, L.; Yang, Y.; Zhi, H.; Liu, X.; Yang, H.;Liu, L., Prediction of Omeprazole Pharmacokinetics and its Inhibition on Gastric Acid Secretion in Humans Using Physiologically Based Pharmacokinetic-Pharmacodynamic Model Characterizing CYP2C19 Polymorphisms. *Pharm Res* **2023,** *40*, 1735-1750.

[3]. Zhou, W.; Johnson, T. N.; Bui, K. H.; Cheung, S. Y. A.; Li, J.; Xu, H.; Al-Huniti, N.;Zhou, D., Predictive Performance of Physiologically Based Pharmacokinetic (PBPK) Modeling of Drugs Extensively Metabolized by Major Cytochrome P450s in Children. *Clin Pharmacol Ther* **2018,** *104*, 188-200.

[4]. Wu, F.; Gaohua, L.; Zhao, P.; Jamei, M.; Huang, S. M.; Bashaw, E. D.;Lee, S. C., Predicting nonlinear pharmacokinetics of omeprazole enantiomers and racemic drug using physiologically based pharmacokinetic modeling and simulation: application to predict drug/genetic interactions. *Pharm Res* **2014,** *31*, 1919-29.

[5]. Qi, F.; Zhu, L.; Li, N.; Ge, T.; Xu, G.;Liao, S., Influence of different proton pump inhibitors on the pharmacokinetics of voriconazole. *Int J Antimicrob Agents* **2017,** *49*, 403-409.

[6]. Katashima, M.; Yamamoto, K.; Sugiura, M.; Sawada, Y.;Iga, T., Comparative pharmacokinetic/pharmacodynamic study of proton pump inhibitors, omeprazole and lansoprazole in rats. *Drug Metab Dispos* **1995,** *23*, 718-23.

[7]. Hanioka, N.; Tsuneto, Y.; Saito, Y.; Maekawa, K.; Sawada, J.;Narimatsu, S., Influence of CYP2C19*18 and CYP2C19*19 alleles on omeprazole 5-hydroxylation: in vitro functional analysis of recombinant enzymes expressed in Saccharomyces cerevisiae. *Basic Clin Pharmacol Toxicol* **2008,** *102*, 388-93.

[8]. Kim, K. A.; Kim, M. J.; Park, J. Y.; Shon, J. H.; Yoon, Y. R.; Lee, S. S.; Liu, K. H.; Chun, J. H.; Hyun, M. H.;Shin, J. G., Stereoselective metabolism of lansoprazole by human liver cytochrome P450 enzymes. *Drug Metab Dispos* **2003,** *31*, 1227-34.

[9]. Steere, B.; Baker, J. A.; Hall, S. D.;Guo, Y., Prediction of in vivo clearance and associated variability of CYP2C19 substrates by genotypes in populations utilizing a pharmacogenetics-based mechanistic model. *Drug Metab Dispos* **2015,** *43*, 870-83.

[10]. Yamazaki, H.; Inoue, K.; Shaw, P. M.; Checovich, W. J.; Guengerich, F. P.;Shimada, T., Different contributions of cytochrome P450 2C19 and 3A4 in the oxidation of omeprazole by human liver microsomes: effects of contents of these two forms in individual human samples. *J Pharmacol Exp Ther* **1997,** *283*, 434-42.

[11]. Hanke, N.; Türk, D.; Selzer, D.; Wiebe, S.; Fernandez, É.; Stopfer, P.; Nock, V.;Lehr, T., A Mechanistic, Enantioselective, Physiologically Based Pharmacokinetic Model of Verapamil and Norverapamil, Built and Evaluated for Drug-Drug Interaction Studies. *Pharmaceutics* **2020,** *12*.

[12]. Qian, C. Q.; Zhao, K. J.; Chen, Y.; Liu, L.;Liu, X. D., Simultaneously predict pharmacokinetic interaction of rifampicin with oral versus intravenous substrates of cytochrome P450 3A/P-glycoprotein to healthy human using a semi-physiologically based pharmacokinetic model involving both enzyme and transporter turnover. *Eur J Pharm Sci* **2019,** *134*, 194-204.

[13]. Ezuruike, U.; Zhang, M.; Pansari, A.; De Sousa Mendes, M.; Pan, X.; Neuhoff, S.;Gardner, I., Guide to development of compound files for PBPK modeling in the Simcyp population-based simulator. *CPT Pharmacometrics Syst Pharmacol* **2022,** *11*, 805-821.

[14]. Shum, S.; Shen, D. D.;Isoherranen, N., Predicting Maternal-Fetal Disposition of Fentanyl Following Intravenous and Epidural Administration Using Physiologically Based Pharmacokinetic Modeling. *Drug Metab Dispos* **2021,** *49*, 1003-1015.

[15]. Guo, H.; Liu, C.; Li, J.; Zhang, M.; Hu, M.; Xu, P.; Liu, L.;Liu, X., A mechanistic physiologically based pharmacokinetic-enzyme turnover model involving both intestine and liver to predict CYP3A induction-mediated drug-drug interactions. *J Pharm Sci* **2013,** *102*, 2819-36.

[16]. Obach, R. S.; Lombardo, F.;Waters, N. J., Trend analysis of a database of intravenous pharmacokinetic parameters in humans for 670 drug compounds. *Drug Metab Dispos* **2008,** *36*, 1385-405.

[17]. Baneyx, G.; Parrott, N.; Meille, C.; Iliadis, A.;Lavé, T., Physiologically based pharmacokinetic modeling of CYP3A4 induction by rifampicin in human: influence of time between substrate and inducer administration. *Eur J Pharm Sci* **2014,** *56*, 1-15.
[18]. Yerasi, N.; Vurimindi, H.;Devarakonda, K., Frog intestinal perfusion to evaluate drug permeability: application to p-gp and cyp3a4 substrates. *Front Pharmacol* **2015,** *6*, 141.
[19]. Acalovschi, M.; Dumitraşcu, D. L.;Csakany, I., Gastric and gall bladder emptying of a mixed meal are not coordinated in liver cirrhosis--a simultaneous sonographic study. *Gut* **1997,** *40*, 412-7.
[20]. Wang, H. Y.; Chen, X.; Jiang, J.; Shi, J.;Hu, P., Evaluating a physiologically based pharmacokinetic model for predicting the pharmacokinetics of midazolam in Chinese after oral administration. *Acta Pharmacol Sin* **2016,** *37*, 276-84.
